# Apigenin: A Bioflavonoid with a Promising Role in Disease Prevention and Treatment

**DOI:** 10.3390/biomedicines12061353

**Published:** 2024-06-18

**Authors:** Khaled S. Allemailem, Ahmad Almatroudi, Hajed Obaid A. Alharbi, Naif AlSuhaymi, Mahdi H. Alsugoor, Fahad M. Aldakheel, Amjad Ali Khan, Arshad Husain Rahmani

**Affiliations:** 1 Department of Medical Laboratories, College of Applied Medical Sciences, Qassim University, Buraydah 51452, Saudi Arabia; k.allemailem@qu.edu.sa (K.S.A.); aamtrody@qu.edu.sa (A.A.); hajed.alharbi@qu.edu.sa (H.O.A.A.); 2 Department of Emergency Medical Services, Faculty of Health Sciences, AlQunfudah, Umm Al-Qura University, Makkah 21912, Saudi Arabiamhsugoor@uqu.edu.sa (M.H.A.); 3 Department of Clinical Laboratory Sciences, College of Applied Medical Sciences, King Saud University, Riyadh 11433, Saudi Arabia; 4 Department of Basic Health Sciences, College of Applied Medical Sciences, Qassim University, Buraydah 51452, Saudi Arabia

**Keywords:** apigenin, inflammation, oxidative stress, pathogenesis, cancer, synergistic effects

## Abstract

Apigenin is a powerful flavone compound found in numerous fruits and vegetables, and it offers numerous health-promoting benefits. Many studies have evidenced that this compound has a potential role as an anti-inflammatory and antioxidant compound, making it a promising candidate for reducing the risk of pathogenesis. It has also been found to positively affect various systems in the body, such as the respiratory, digestive, immune, and reproductive systems. Apigenin is effective in treating liver, lung, heart, kidney, neurological diseases, diabetes, and maintaining good oral and skin health. Multiple studies have reported that this compound is capable of suppressing various types of cancer through the induction of apoptosis and cell-cycle arrest, suppressing cell migration and invasion, reduction of inflammation, and inhibiting angiogenesis. When used in combination with other drugs, apigenin increases their efficacy, reduces the risk of side effects, and improves the response to chemotherapy. This review broadly analyzes apigenin’s potential in disease management by modulating various biological activities. In addition, this review also described apigenin’s interaction with other compounds or drugs and the potential role of nanoformulation in different pathogeneses. Further extensive research is needed to explore the mechanism of action, safety, and efficacy of this compound in disease prevention and treatment.

## 1. Introduction

It is recognized that traditional medicines based on plant sources have been used for centuries to treat various diseases. Moreover, natural products are a substantial source of new compounds for drug research as well as development [1].

**Figure 1:**
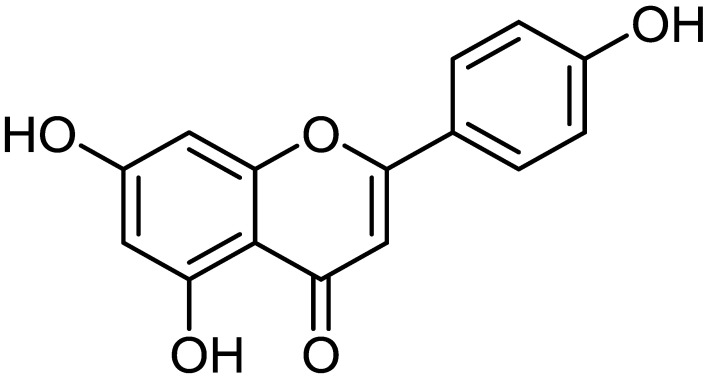
Chemical structure of apigenin (the structure was drawn using ChemDraw professional 15.0).

Flavonoids are natural ingredients with variable phenolic structures that are found in vegetables, fruits, stems, roots, grains, bark, tea, and flowers [2]. *Apigenin* (4′,5,7-trihydroxyflavone) (Figure 1) is a flavonoid present in fruits as well as vegetables, belonging to the flavone class that is the aglycone of several naturally occurring glycosides; its melting point is 347.5 °C, its chemical formula is C_15_H_10_O_5_, and its molecular weight is 270.24 [3,4]. It has three hydroxyl groups, with the first as well as second in the C5 and C7 positions, and the third at C4′ of the B ring [5].

In addition, the aglycone, other forms are glycosylated derivatives (apigenin apiin), methylated derivatives (apigenin-5,7,4′-trimethyl ether and apigenin-7,4′-dimethyl ether), sulfated derivatives (e.g., apigenin-7-sulfate), and dimers including amentoflavone (3′,8″-biapigenin) of apigenin have been described [6,7]. Moreover, another methoxy derivative of apigenin is acacetin (Apigenin-7-O-β-D-galactopyranoside), and acacetin is extracted from *Turnera diffusa* and *Chrysanthemum morifolium* [8].

The other common apigenin glycosides are apigenin-7-O-glucoside, apigenin-8-C-glucoside (vitexin), apigenin-6-C-glucoside (isovitexin), and apigenin-7-O-neohesperidoside (rhoifolin), as well as apigenin-6-C-glucoside-8-C-arabinoside [9].

Apigenin is lipophilic and may be inactivated in the acidic environment of the gastrointestinal tract, resulting in reduced bioavailability. This limits its potential use in healthcare products and functional foods [10]. It has been stated to be practically insoluble/poorly soluble in water [11,12]. Due to its poor water solubility, its oral bioavailability is poor [12]. The room temperature solubilities of apigenin in different solvents and surfactants are analyzed in the order of decreasing solubility, along with the solubility parameter and hydrophilic–lipophilic balance (HLB) values. Apigenin was more soluble in DMSO (>100 mg/mL) than in any other solvents examined. Apigenin was also soluble to some extent in acetone and alcohols (PG; 1.02–1.63 mg/mL). Moreover, this compound was practically not soluble in highly polar solvents, such as water (0.00135 mg/mL), and nonpolar solvents, including silicon fluid (0.0728 mg/mL) and safflower oil (0.0317 mg/mL) [13].

Apigenin mainly plays a role as an anti-inflammatory substance and can regulate the production and gene expression of mucin via regulating NF-κB signaling pathways in airway epithelial cells [14]. Moreover, apigenin is an abundant source of antioxidants, and this compound ameliorates oxidative stress and mitochondrial damage induced by multiwall carbon nanotubes in rat kidney mitochondria [15].

Previous studies based on in vivo and in vitro research reported that this compound has the potential to suppress various types of cancers through the induction of apoptosis and cell-cycle arrest, suppressing cell migration and invasion, and inhibiting angiogenesis [16]. This review comprehensively analyzes apigenin’s role in health management by modulating biological activities. It also discusses its interaction with other compounds/drugs and its potential role in pathogenesis.

## 2. Sources, Intake, and Safety of Apigenin

**Figure 2:**
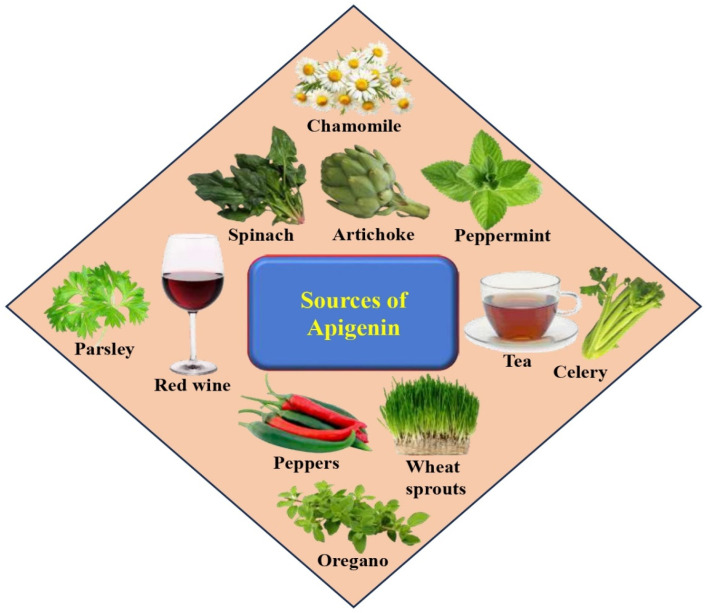
Some dietary sources of apigenin.

Flavonoids are a diverse group of naturally occurring polyphenolic compounds synthesized by plants. They have a similar chemical structure and are classified into different subclasses including anthocyanidins, flavanols, flavanones, flavonols, flavones, and isoflavones, among others [17]. *Apigenin* belongs to the flavone subclass of flavonoids; several vegetables (beans, tomatoes, spinaches, artichokes, celery, parsley, onions), fruits (cherries, apples, grapes, oranges), herbs (chamomile, thyme, peppermint, oregano, basil, marjoram), and plant-based beverages (tea, beer, wine) are good sources of apigenin (Figure 2). Moreover, several foods are rich in apigenin, including celery seed, fresh parsley, dried oregano, vine spinach, green celery heart, and Chinese celery [18]. Dried parsley has a principally high level of apigenin that far exceeds any other herbs or vegetables [18]. Good sources of apigenin include Chinese cabbage (187.0 mg/kg), French peas (176.0 mg/kg), and bell pepper (272.0 mg/kg). Moreover, wolfberry leaves (547.0 mg/kg), belimbi fruit (458.0 mg/kg), garlic (217.0 mg/kg), snake gourd (42.4 mg/kg), guava (579.0 mg/kg), local celery (338.5 mg/kg), and kadok (34.5 mg/kg) as well as daun turi (39.5 mg/kg), are all good sources [19]. The presence of apigenin in alcoholic beverages, such as red wine and beer. Apigenin is normally found as a constituent in red wine [20], and beer also offers a good source of apigenin [21].

Moreover, celery is also a good source of apigenin, holding 108 mg of apigenin per kg [22,23]. Another source of apigenin consumed as a single ingredient in herbal tea is chamomile, prepared from the dried flowers of *Matricaria chamomilla* [24]. Another finding reported that apigenin was found in Swedish turnip (154.0 mg kg^−1^), celery leaves (248.0 mg kg^−1^), and celery root (24.1 mg kg^−1^) [25]. Furthermore, in a Danish survey of celery leaves, apigenin (740 mg kg^−1^) was found, and in parsley, apigenin (1850 mg kg^−1^) was also detected [26].

Studies have shown that a diet high in flavonoid-rich foods, such as fruits, vegetables, and red wine, is linked to a lower risk of overall mortality, mortality from coronary heart disease, and a reduced risk of cancer and Alzheimer’s disease [27,28,29]. Apigenin, a flavonoid found in various fruits, vegetables, and plant-based beverages, reduces pathogenesis development.

A more detailed assessment of daily dietary flavonoid intake found that 1 g can be consumed as glycosides or 650 mg can be consumed as aglycones [30]. Another study reported an average of only 23 mg/day in adults in the Netherlands [31]. In the bioavailability study conducted by Meyer and colleagues, adult volunteers consumed a meal containing 2 g of parsley per kilogram of body weight. This amount corresponded to 149 ± 35 g of parsley (mean body weight 75 kg) and provided an average of 18 ± 4 mg (66 ± 15 μmol) of apigenin [32]. Observational studies have shown that the daily intake of apigenin within whole foods in the human diet varies between 0.45 and 1.17 mg, depending on age and demographic factors [32,33].

In 1992, the average daily intake of the flavones apigenin and luteolin was 1 mg each [31]. Another study was performed based on China to evaluate the relationship between the intake of different flavonoids and their corresponding plasma concentrations. The mean intake estimate of apigenin was 4.23 mg/day [34].

The safety and toxicity levels of any bioactive compound or flavonoid are crucial to consider before using them. This helps ensure the safety of the compound for human consumption or other applications. Generally, apigenin is considered safe and does not cause severe toxicity even at higher doses. The evaluation of the acute toxicity of apigenin showed no mortality or signs of toxicity in mice or rats at oral doses up to 5000 mg/kg [35]. Furthermore, in vitro assessment demonstrated that apigenin has no carcinogenic or mutagenic effects [36,37]. Moreover, at high doses, it can trigger sedation and muscle relaxation [38].

## 3. Exploring the Pharmacological Potential of Apigenin through the Modulation of Biological Activities

### 3.1. Oxidative Stress

**Figure 3:**
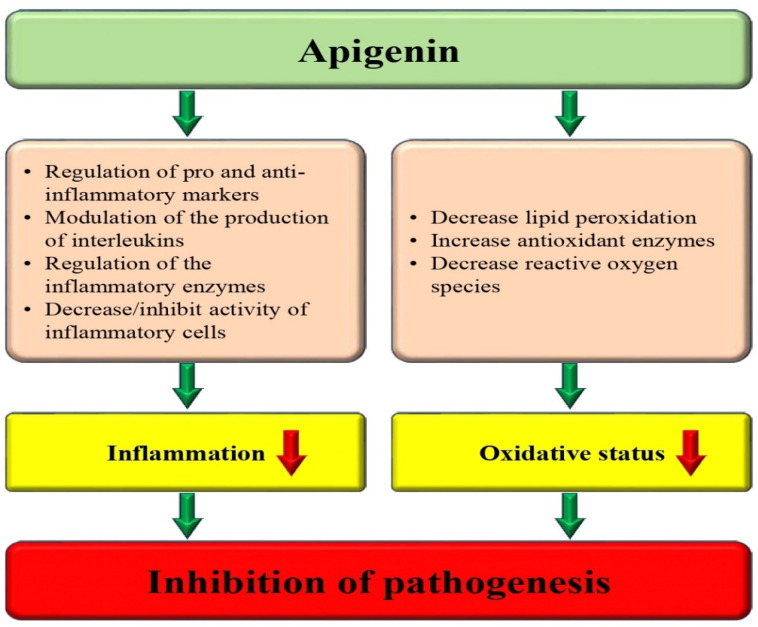
Role of apigenin in disease management through inhibition of oxidative stress and inflammation. The downward pointing arrow signifies downregulation.

Oxidative stress is a condition where there is an imbalance between the production and degradation of reactive oxygen species (ROS) or reactive nitrogen species (RNS) [39]. ROS are involved in various cellular processes, like cell proliferation, differentiation, and death in different cell types [40,41]. However, they can also cause damage to many biomolecules through specific processes, such as pro-inflammatory cytokine secretion and lipid peroxidation [41]. This can cause damage to cells and has been linked to various health issues. However, natural compounds, like polyphenols and flavonoids, have been shown to have potent antioxidant properties that can help neutralize these free radicals and prevent damage [42,43]. Apigenin is a flavonoid that has been revealed to keep antioxidant potential, which may help to scavenge free radicals reduce oxidative stress, and finally inhibit various pathogenesis (Figure 3). According to the results, the study suggests that apigenin could potentially help alleviate age-related skeletal muscle atrophy. This could be due to its ability to reduce oxidative stress and inhibit hyperactive autophagy and apoptosis [44]. The study observed that when osteoblastic cells were treated with H_2_O_2_, apigenin had a protective effect. However, pretreatment of cells with apigenin reduced all the H_2_O_2_-induced effects. This compound increased the expression of certain antioxidant enzymes, specifically SOD1, SOD2, and GPx1. Based on the outcomes, this study proposes that apigenin could potentially attenuate oxidative-induced cell damage in osteoblastic cells [45]. The activity of apigenin in scavenging reactive carbonyl species (RCS) and the molecular mechanism involved in its protective consequence against advanced glycation end products (AGEs)-induced oxidative stress along with inflammation were investigated. It is interesting to note that apigenin (API) has the ability to directly trap methylglyoxal (MGO) and form API-MGO adducts. This mechanism has been found to inhibit the formation of advanced glycation end products (AGEs). Studies have found that both API and di-apigenin adducts have the ability to inhibit advanced glycation end products (AGEs)-induced oxidative stress and inflammation in human umbilical vein endothelial cells [46]. The role of apigenin in doxorubicin (DOX)-induced oxidative injury was investigated. Certainly, SOD activity decreased in the DOX group and recovered in the apigenin groups. The DOX-induction caused a higher level of MDA and the reduced GSH was upregulated by apigenin. Furthermore, it was reported that apigenin rescued the DOX-induced drop in SOD2 in the kidney. DOX increased the generation of ROS and superoxide in a time-dependent way. However, APG meaningfully suppressed the overproduction of DOX-induced ROS induced by DOX. These findings proposed that apigenin suppressed oxidative stress caused by DOX [47]. The potential role of apigenin in the inhibition of oxidative stress-initiated melanocyte reduction in vitro via a PIG3V vitiligo perilesional melanocyte cell model has been examined. Findings revealed that as compared with negative control cultures, apigenin-treated cells had increased viability. Similarly, apigenin enhanced the expression of the cellular antioxidants SOD, GSH-Px, and CAT, but prevented MDA production. Remarkably, the expression and nuclear localization of the Nrf2 transcription factor, an important regulator of oxidative stress, was meaningfully increased by apigenin treatment [48]. The apigenin relieves muscle atrophy in aged mice, possibly through special effects on reactive oxygen species; as such, enzymes with antioxidant functions were examined. The findings of the study proposed that apigenin endorsed the activities of enzymes, including superoxide dismutase as well as glutathione peroxidase for antioxidation. Finally, the results suggest that apigenin relieves age-linked skeletal muscle atrophy via decreasing oxidative stress and inhibiting hyperactive autophagy and apoptosis [44]. This study aimed to evaluate whether apigenin alleviates early brain injury (EBI) after subarachnoid hemorrhage (SAH) through its anti-oxidative and anti-apoptotic effects. Apigenin treatment caused a reduction in the concentration of malondialdehyde (MDA), reactive oxygen species (ROS), and myeloperoxidase (MPO), elevated the ratio of oxidized glutathione and glutathione, and increased the amount of superoxide dismutase in brain cortex at 24 h following SAH [49]. 

### 3.2. Anti-Inflammatory Potential

Inflammation can be a common factor in the development of various chronic diseases, such as neurodegenerative disorders, bowel diseases, cardiovascular diseases, arthritis, cancer, and diabetes [50]. The excessive over-expression of cytokine signaling can play a significant role in the complexity of various disease conditions [51]. The anti-inflammatory potential of apigenin has been reported through the reduction in inflammatory markers/enzymes (Table 1 and Figure 3). Inflammatory cytokine production was evaluated to measure the anti-inflammatory properties of apigenin. Lipopolysaccharide (LPS) caused an important increase in IL-1ß, TNF-a, and IL-6 production both in mRNA and in protein levels. Apigenin meaningfully suppressed the production of these inflammatory cytokines compared to the LPS group [52]. A study result demonstrated that administration with apigenin increased Th1 cytokine and transcription factor levels and decreased Th2 cytokine and transcription factor levels and promoted the ratio of Th1/Th2 cells in allergic rhinitis mice [53]. The objective of the study was to investigate the anti-inflammatory effects of apigenin to evaluate its potential as an anti-psoriatic agent. According to the study, apigenin is effective in downregulating the expression and secretion of pro-inflammatory cytokines through the IL-23/IL-17/IL-22 axis. It has been observed that apigenin can suppress the nuclear translocation of NF-κB in LPS-induced RAW 264.7 cells [54]. Sepsis led to an increase in TNF α, IL-1-β, and IL-6 levels. Apigenin (20 and 40 mg/kg) reduced these pro-inflammatory cytokine levels [55]. The study examined the effects of apigenin on the gene expression and protein secretion of TNF-α and IL-10 in RAW-264.7 cells. Apigenin at a dose of 30 μM significantly decreases the IL-10 and TNF-α expression and secretion [56]. 

Earlier research has revealed that apigenin possesses anti-inflammatory properties that can help alleviate skin inflammation. This is achieved by downregulating the expression of cyclooxygenase-2 (COX-2) [30,57]. In an NC/Nga mouse model, it was observed that apigenin reduced the levels of IgE and interferon (IFN)-γ in serum [58]. The anti-inflammatory effect of apigenin was investigated in an experimental model of acute pancreatitis. In the control group, there was an over-expression of TNF-α in relation to the postoperative time. However, in the apigenin group, there was an under-expression of TNF-α during the postoperative time. At 72 h, it was observed that apigenin reduced pancreatic TNF-α expression and prevented pancreatic necrosis [59]. According to another study, apigenin was found to effectively inhibit the production of pro-inflammatory cytokines, like IL-1β, IL-6, and TNF-α, in macrophages that were induced by LPS. This was accomplished through the modulation of various intracellular signaling pathways [60]. Apigenin showed anti-inflammatory activity that involves blocking NO-facilitated COX-2 expression as well as monocyte adherence [61].

### 3.3. Anti-Diabetic Potential

**Figure 4:**
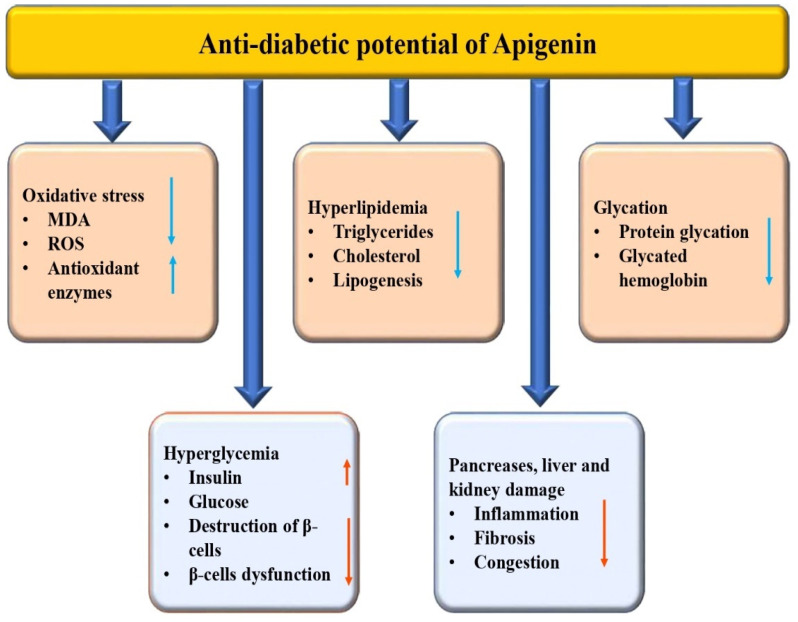
The anti-diabetic potential of apigenin through the modulation of various biological activities. The upward pointing arrow signifies upregulation and the downward pointing arrow signifies downregulation.

Diabetes mellitus is a medical condition characterized by abnormally high blood glucose levels caused by either insufficient insulin production, insulin resistance, or a combination of both [62]. There are several natural compounds and bioactive compounds that have been studied for their potential role in managing diabetes mellitus and its complications [63,64,65]. There is a growing body of research that supports the use of traditional medicinal plants for the management of diabetes. Several plants have been found to have hypoglycemic and antidiabetic effects, and the mechanisms of their activity have been studied extensively [66,67,68]. The modulation of various biological activities has led to reports of the anti-diabetic potential of apigenin in previous studies (Table 1 and Figure 4). In diabetic rats, treatment with 20 mg/kg of apigenin resulted in a significant attenuation of renal dysfunction, oxidative stress, and fibrosis. Apigenin’s prevention of MAPK activation is noteworthy as it inhibits inflammation by reducing TNF-α, IL-6, and NF-κB expression, and also contributes to a reduction in apoptosis. Additionally, histopathological findings confirmed reduced inflammation, glomerulosclerosis, and collagen deposition in the renal tissue [69]. A study was performed to examine the roles of apigenin in cardiac remodeling of diabetic cardiomyopathy. The findings of that study suggested that diabetes mellitus can worsen cardiac dysfunction, leading to an increase in the accumulation of 4-hydroxynonenal, fibrosis, and a decrease in the expression of Bcl2, GPx, and SOD [70]. A previous study reported that the administration of apigenin to diabetic animals increased the levels of thyroid hormones and serum insulin and decreased glucose concentration as well as hepatic G-6-Pase activity [71]. Compared to the diabetic control group, apigenin, and naringenin significantly reduced blood glucose, serum lipid, malondialdehyde, ICAM-1, and insulin resistance index levels. Moreover, the apigenin group caused an increased SOD activity as well as improved impaired glucose tolerance [72].

### 3.4. Hepatoprotective Effects

**Figure 5:**
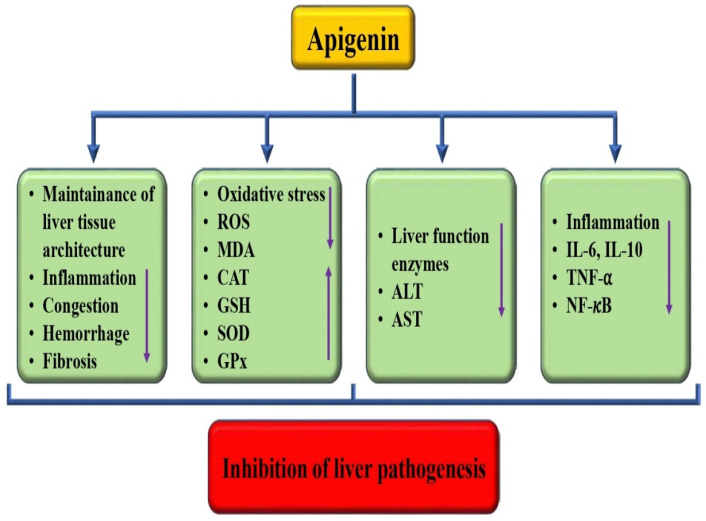
Hepatoprotective/inhibition of liver pathogenesis potential of apigenin through the regulation of various biological activities. The upward pointing arrow signifies upregulation and the downward pointing arrow signifies downregulation.

Liver-associated pathogenesis is increasing worldwide and is the main cause of mortality. The current mode of treatment used in this pathogenesis causes adverse effects. Natural compounds or their bioactive compounds can protect the liver by reducing inflammation and oxidative stress and by improving the tissue architecture [73,74]. The hepatoprotective role of apigenin has been described in previous studies (Table 1 and Figure 5). The hepatoprotective potential of apigenin has been reported by previous studies through the regulation of various biological activities. A study reported that in CCl4-treated group mice, multifocal hepatic parenchymal necrosis was seen. However, apigenin reduced the pathological changes. Moreover, it was indicated that increased serum AST and ALT levels were observed in CCl4-treated mice. However, apigenin reversed such changes, which suggested that apigenin could prevent liver damage caused by CCl4. Apigenin restores the antioxidant activity and decreases lipid peroxidation, thus inhibiting oxidative stress caused by CCl4. Moreover, apigenin’s role as an anti-inflammatory was noticed, as the IL-6, IL-10, and TNF-α content in the model group increased [75]. Ali et al. conducted a study that showcased apigenin pretreatment for alleviating the effects of NiONPs. The pretreatment was found to be able to prevent oxidative stress, fibrosis, and inflammation [76]. Another study reported that apigenin played a crucial role in improving hepatic function by reducing the activity of alanine aminotransferase, as well as decreasing serum dyslipidemia, such as LDL cholesterol and total cholesterol levels. Additionally, this compound was found to lower lipid peroxidation and oxidative stress capacity while enhancing the activities of superoxide dismutase and GSH peroxidase [77]. Based on the findings, it was observed that mice treated with apigenin showed a decrease in the levels of hepatic malondialdehyde and tumor necrosis factor-alpha [78]. Another study was made to explore the effects of apigenin on acute liver injury. The results indicated that apigenin pretreatments led to a decline in the liver injury indices of oxidative stress as well as inflammatory events and that this compound possibly has liver-protective effects in liver injury [79]. Cells treated with various concentrations of apigenin reduced palmitic acid-induced increases in total cholesterol, triglyceride levels, and lipid accumulation, according to reports [80].

### 3.5. Renoprotective Effects

Kidney-associated pathogenesis poses a significant health burden worldwide. The renoprotective potential of apigenin has been reported by previous studies (Table 1). The researchers carried out a study to investigate the potential nephroprotective effects of apigenin as a dietary supplement against renal injury caused by cisplatin. They used human embryonic kidney cells as their in vitro model for the study. According to the results of the study, the combination of CIS 11.36 µM + API 12.5 µg/mL was found to be effective in protecting against the nephrotoxicity induced by cisplatin [81]. The experiment was performed to explore the potential effect of a plant flavone called apigenin in mitigating the nephrotoxic effects induced by cyclosporine. It was noticed that renal damage was noticed by the treatment of cyclosporine alone. Furthermore, blood urea nitrogen, uric acid, urea, and lipid hydroperoxides were increased while there was a noteworthy decrease in the total antioxidant levels. The study found that treatment with apigenin resulted in a significant reduction in lipid hydroperoxides and an increase in total antioxidant levels. Moreover, concurrent treatment with apigenin was found to significantly reduce the histopathological changes observed in the groups treated with cyclosporine [82]. It seems that apigenin had a positive effect on reducing renal function markers, such as serum creatinine and urea nitrogen content. Additionally, it appears that apigenin was able to restore some of the renal tissue lesions that were caused by 3-MCPD treatment [83]. The study aimed to investigate the potential antiapoptotic effects of apigenin on human renal proximal tubular epithelial cells (HK-2) that were treated with cisplatin. It is interesting to note that the research findings suggest apigenin has a cytostatic activity by inducing cell cycle arrest. Additionally, it was observed that apigenin inhibited the cisplatin-induced apoptosis of HK-2 cells. Moreover, apigenin was also found to inhibit caspase-3 activity and PARP cleavage in cisplatin-treated cells [84]. Liu et al. (2017) demonstrated that pretreating rats with apigenin for 24 h provided protection against renal ischemia followed by reperfusion [85].

The administration of nickel oxide nanoparticles (NiONPs) alone caused significant disturbances in the kidney as well as liver tissues. Subcellular changes were also noted in these organs using TEM. However, administering apigenin before the administration of nickel oxide nanoparticles (NiONPs) showed significant alleviation of all the studied parameters, indicating a potential protective effect of apigenin on liver and kidney tissues [76]. A recent study has shown that multiwall carbon nanotubes can enter the human body and cross cellular barriers, reaching sensitive organs, such as the kidneys. This can cause damage to mitochondria in kidney cells, specifically renal tubular cells. Apigenin can effectively treat multiwall carbon nanotube-induced kidney damage [15].

### 3.6. Cardioprotective Effects

**Figure 6:**
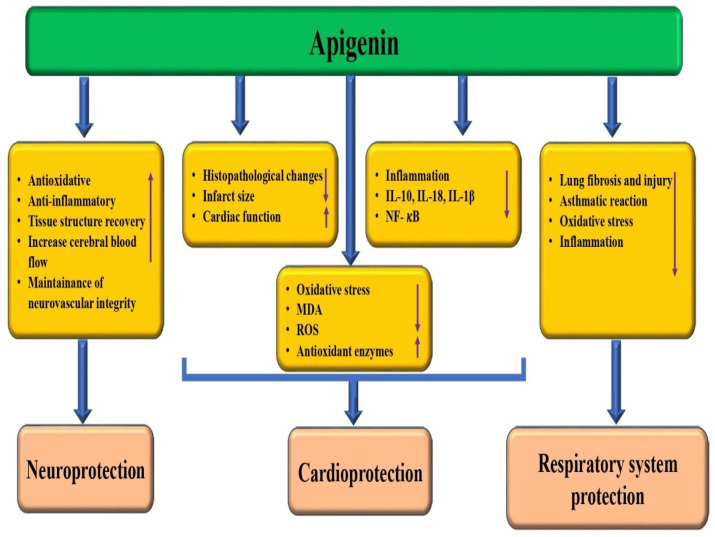
Apigenin plays cardio-, neuro-, and respiratory system-protective roles through the modulation of various biological activities. The upward pointing arrow signifies upregulation and the downward pointing arrow signifies downregulation.

Natural compounds/bioactive compounds and their potential roles in protecting the pathogenesis through different mechanisms. Apigenin plays a role as a cardioprotective substance through the modulation of various biological activities (Figure 6). Study results found that apigenin-7-O-β-D-(6″-p-coumaroyl)-glucopyranoside was effective in reducing myocardial infarct size in an experimental model of myocardial ischemia/reperfusion injury. The administered apigenin showed significant reductions in myocardial infarct size. Furthermore, this compound not only decreases the myocardial infarct size but also suppresses myocardial injury enzymes and pro-inflammatory cytokines [86], and apigenin attenuated the apoptosis and pyroptotic myeloblastosis of H9c2 cells induced by I/H injury [87]. Apigenin pretreatment showed protective effects on the myocardium against I/R injury by improving cardiac function, reducing infarct size, and decreasing the LDH and CPK activities. However, these protective effects were abrogated by GSI. Apigenin pretreatment protected against SI/R injury in the isolated hearts via reducing apoptosis, which was reversed by GSI, and pretreatment with this compound upregulated Hes1 expression [88]. Apigenin treatment improved left ventricular function and redox balance and prevented hemodynamic perturbations [89]. Another important study reported that apigenin treatment prevented isoproterenol hydrochloride-induced lipid peroxidative levels and antioxidant status in cardiomyoblast H9C2 cells. Additionally, apigenin prevented the expression of inflammatory markers in isoproterenol hydrochloride-treated cells [90]. It has been found that intragastric administration of apigenin (25 mg/kg) can reduce myocardial damage, and that it can significantly improve cardiac functional parameters [91].

### 3.7. Neuroprotective Potential

Apigenin plays a role as a neuroprotective through the modulation of various biological activities (Figure 6). A study was carried out to focus on evaluating the potential of apigenin as a neuroprotective and neuro-immunomodulatory agent using in vitro models of neuroinflammation associated with Alzheimer’s disease. The results of the study showed that apigenin treatment preserved neurons as well as astrocyte integrity and that this compound was not neurotoxic and has a neuroprotective property against inflammatory damage [92]. The neuroprotective potential of apigenin has been reported by previous studies (Table 1). An interesting research study looked into the potential of apigenin to improve neonatal hypoxic–ischemic (HI) brain injury. The study used in vivo experiments to explore the mechanisms associated with this. Apigenin significantly reduced the infarct volume, decreased the inflammatory response, ameliorated cerebral edema, inhibited apoptosis, promoted tissue structure recovery, and improved prognosis following brain injury. Also, it was found that apigenin showed a neuroprotective property against HI brain injury [93]. A study was conducted to explore the potential benefits of apigenin in reducing inflammation, providing antioxidant effects, and protecting the brain after a mild traumatic brain injury (TBI). The study used a TBI model to explore the potential benefits of apigenin. After TBI, lucigenin and luminol levels were enhanced, and lucigenin and luminol levels diminished with treatments of apigenin. In the context of trauma, the levels of interleukin, which is an anti-inflammatory cytokine, tend to decrease. However, treatment with apigenin at doses of 20 and 40 mg has been shown to increase the levels of interleukins. The histological damage score in the cortex was reduced in the apigenin treatment (20 mg) group [94]. Apigenin treatment has been found to have a positive impact on mitigating the effects of spinal cord injury. Specifically, it was found to reverse the reduction of antioxidant enzyme activity, while also countering the increase in the MDA level caused by the injury. After spinal cord injury, apigenin treatment leads to a decline in the release of serum interleukin-1β, tumor necrosis factor-α, and intercellular adhesion molecule-1, indicating anti-inflammatory effects. The data obtained from the study provide strong evidence that apigenin plays a significant role in promoting the recovery of rat neuronal function after spinal cord injury. This is attributed to its various properties, including antioxidative, anti-inflammatory, and antiapoptotic properties [95]. The oral administration of apigenin has been shown to confer neurovascular coupling protection. This protection involves improvements to memory capabilities, modulation of microvascular function, and the maintenance of neurovascular unit integrity [96].

### 3.8. Role in the Respiratory System 

Pathogenesis related to the respiratory system is a substantial reason for mortality globally. Medicinal plants and their bioactive compounds have been found to play a role in the management of lung pathogenesis. This is achieved by regulating oxidative stress, inflammation, fibrosis, and lung tissue injury [97,98,99,100]. The use of natural remedies derived from plants may offer a promising alternative approach to treating lung diseases. That apigenin plays a role in the respiratory system has been evidenced (Figure 6 and Table 1). The study aimed to examine the potential impact of apigenin on lung fibrosis induced by bleomycin in rats. The results of the study showed that oral administration of apigenin prevented the fibrotic process induced by bleomycin. The treatment was found to suppress the increases in tumor necrosis factor-α, myeloperoxidase activity, and transforming growth factor-β level and to attenuate the reduction in antioxidant enzyme activity caused by bleomycin [101]. The study was designed to explore the potential of apigenin, a natural flavonoid, in reducing airway inflammation in asthmatic mice exposed to PM2.5. Apigenin treatment reduced airway hyper-responsiveness, eosinophil percentage, as well as neutrophil infiltration in OVA-sensitized, BALF, and PM2.5-exposed mice [102]. Ovalbumin (OVA)-induced mice exhibited various allergic airway reactions. Additionally, administration of apigenin before the last airway OVA challenge caused a noteworthy inhibition of all asthmatic reactions [103]. It appears that after being stimulated with lipopolysaccharide (LPS) for six hours, there were changes in various aspects of the lungs, including myeloperoxidase activity, cytokines in bronchoalveolar lavage fluid, pulmonary pathological features, total polymorphonuclear leukocytes (PMN) cells, and airway oxidative stress enzymes. Apigenin-7-glycoside inhibited the LPS-enhanced inflammatory activity in the lung and also showed an anti-inflammatory effect via the MAPK and inhibitor NF-κB (IκB) pathways [104]. Apigenin was found to decrease the degree of airway hyper-responsiveness and inflammatory cell infiltration when compared to the ovalbumin group [105].

### 3.9. Role in the Reproductive System

A study examined the potential protective effects of apigenin against acrylonitrile-induced subchronic sperm and testes injury in rats. Findings revealed that this compound increased sperm concentration, mitochondrial membrane potential, and motility, which were reduced by acrylonitrile. On the other hand, malondialdehyde and reactive oxygen species were significantly decreased by apigenin. Apigenin decreased spermatogenic cell apoptosis and pathological injuries caused by acrylonitrile in rat testes [106]. Another study result indicated that sperm density was reduced in the 25 mg/kg group as compared to the control group. Moreover, the percentage of seminiferous epithelium cells at the cell-circle phase of G0/G1 showed a noteworthy increase in the 25 mg/kg group as compared with the control groups [107]. Research based on mice was carried out to evaluate the role of apigenin on semen parameters. At 14 days, the high-dose apigenin group showed outstanding decreases in average path velocity, straightness, straight-line velocity, wobbliness, sperm motility, and the percentage of grade b sperm, and a significant increase in beat cross frequency as compared with the negative control group [108]. Another finding revealed that apigenin has a substantial ability to exert a dual-directional estrogenic effect. It has also been observed that this flavonoid has noteworthy estrogenic activity, as evidenced by its ability to reverse uterine atrophy. Additionally, apigenin treatment has been observed to regulate target tissue coefficient changes and address estrogen disorders caused by excessive estrogen levels [109]. A recent study result reported that cyclophosphamide-intoxicated mice caused low sperm count, motility viability, and testosterone levels. The administration of apigenin attenuated testosterone levels as well as sperm measurements. This compound administration reduced the CP-induced testicular damage via increasing antioxidants, suppressing the pro-inflammatory cytokines and decreasing apoptosis [110].

### 3.10. Role in Skin Disease

**Figure 7:**
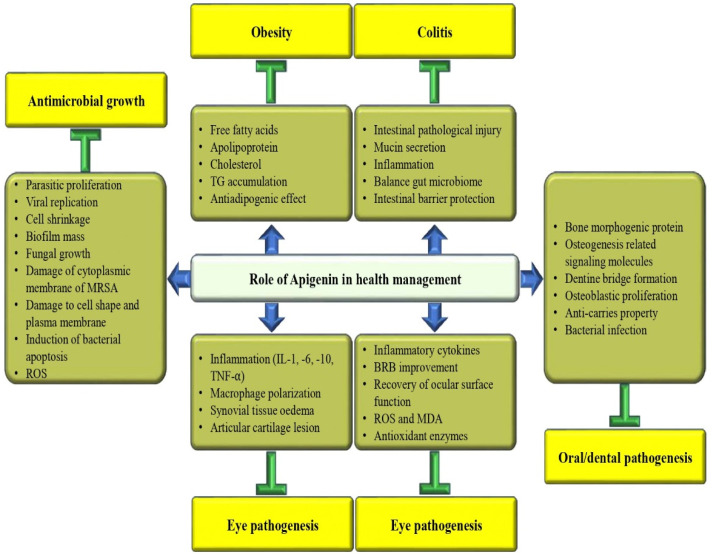
Role of apigenin in different pathogeneses.

Apigenin, a flavonoid, shows a role in skin disease via the modulation of various biological activities (Figure 7). As per the study findings, it was observed that upon irradiation of cells with 25 J/cm^2^ ultraviolet light, the percentage of senescent cells increased significantly to 61.29%. However, upon subsequent treatment with apigenin at 5, 10, and 20 µM concentrations, a dose-dependent decrease in the number of senescent cells was observed. The percentage of senescent cells was reduced to 50.49%, 32.03%, and 17.34%, respectively. An in vivo study was conducted to evaluate the impact of apigenin on skin aging. The results showed that those who used a cream without apigenin showed a mean density of 50.41 µm before use, and densities of 50.02 and 50.05 µm after 2 and 4 weeks, respectively. However, the group using the apigenin-containing cream a saw significant improvement, with a mean density of 49.96 µm before use and impressive increases of 55.31 µm and 62.32 µm after 2 and 4 weeks of use, respectively. Moreover, the topical application of apigenin improved skin elasticity. According to the study, the application of the cream containing apigenin resulted in a significant improvement in skin moisture levels. The results showed a 32.36% and 51.38% increase in skin moisture after 2 and 4 weeks of use, respectively [111]. Furthermore, apigenin showed a reduction in the number of cyclobutane pyrimidine dimers (CPDs) after 24 h. Thus, apigenin enhanced the reversal of UV-B-induced CPDs via upregulation of NER genes, inhibition of ROS generation, and downregulation of NF-κB and MAPK [112]. Based on in vivo skin model studies, apigenin has been reported to be highly powerful in inhibiting the development of UVB-induced ear edema, as well as decreasing COX-2 expression in SKH-1 hairless mice [113]. The results of the study reported that the use of topical apigenin can deliver therapeutic benefits for allergic contact dermatitis and acute irritant contact dermatitis. Moreover, when compared to the vehicle treatment, applying apigenin topically showed significant reductions in transepidermal water loss and skin surface pH in the allergic contact dermatitis model [114]. Topical apigenin application can improve the function of the skin’s permeability barrier by promoting various processes including epidermal differentiation, cutaneous antimicrobial peptide production, and lipid synthesis and secretion. This could have potential benefits for the prevention as well as treatment of skin disorders [115].

### 3.11. Anti-Obesity Potential

Apigenin plays a role in anti-obesity potential through the modulation of various biological activities (Figure 7). An important study result reported that apigenin reduces body weight in high-fat diet (HFD)-induced obese mice. Furthermore, a HFD increases STAT3 phosphorylation in VAT, although not in EAT and SAT. Additional studies suggest that apigenin binds to non-phosphorylated STAT3 and decreases STAT3 phosphorylation as well as transcriptional activity in VAT [116]. According to a recent study, a researcher has investigated the possible protective effects of apigenin in HFD-induced obese mice against obesity as well as related metabolic issues. It is interesting to note that in HFD-fed mice, apigenin appears to have had positive effects on various markers of metabolic health. These included dropping plasma levels of free fatty acid, total cholesterol, hepatic dysfunction markers, and apolipoprotein [117]. The research has shown that apigenin treatment can alleviate issues, such as body weight, glycolipid metabolic disorder, and insulin resistance in mice [118]. Apigenin has been found to protect dibutyryl-cAMP-induced browning from IL-1β in primary human adipocytes. This has been evidenced by an increase in brown-specific markers, oxygen consumption, and mitochondrial content. This compound has been found to significantly repress inflammatory markers. Interestingly, apigenin also profoundly induces cyclooxygenase 2 as well as prostaglandin E2 expression in response to IL-1β [119]. The study involved culturing pre-adipocytes from day zero to day eight, followed by mature adipocytes for forty-eight hours. The cells were treated with different doses of polyphenols (apigenin, hesperidin, and kaempferol)—1 µM, 10 µM, and 25 µM—to investigate their effects on the cells. The three polyphenols were found to reduce TG accumulation in mature adipocytes. Moreover, apigenin as well as hesperidin were found to decrease FASN expression [120].

### 3.12. Anti-Depressive Effects

The anti-depressive activity of apigenin was measured in mice, and treatment with this flavonoid reduced anxiety and immobility time and reversed anhedonia in behavioral studies. Furthermore, apigenin enhanced levels of antioxidant enzymes and reduced malondialdehyde and corticosterone levels. This compound has shown potential in attenuating interleukin-6 and *TNFα*, as well as in restoring cell loss [121]. In the STZ-mediated depression model, the study found that a dose of 20 mg/kg of apigenin was effective in minimizing negative side effects. The data further suggested that apigenin has the potential to regulate behavioral dysfunction, biochemical biomarkers, and cellular antioxidant levels in depressed animals [122]. The study investigated the potential antidepressant effects of apigenin on mice with depression-like symptoms through the regulation of autophagy. According to the study, the expression levels of LC3-II/I and p62 were used to determine the autophagy levels. The results showed that apigenin treatment was able to significantly increase the inhibited autophagy level induced by chronic restraint stress. The study’s findings suggest that apigenin may promote autophagy in mice with chronic restraint stress-induced depression via the AMPK/mTOR pathway [123]. To inspect the neurotrophic-related mechanism of this compound in mice with depressive symptoms caused by corticosterone treatment, a study was conducted. The mice were subjected to repetitive injections of corticosterone at a dose of 40 mg/kg subcutaneously once daily for 21 consecutive days. To examine the effects of apigenin as well as fluoxetine, they were administered 30 min before the corticosterone injection at doses of 20 and 40 mg/kg and 20 mg/kg, respectively. The study advises that the antidepressant-like role of apigenin may be partially attributed to the increase in brain-derived neurotrophic factor levels in the hippocampus [124].

### 3.13. Anti-Cancer Potential

**Figure 8:**
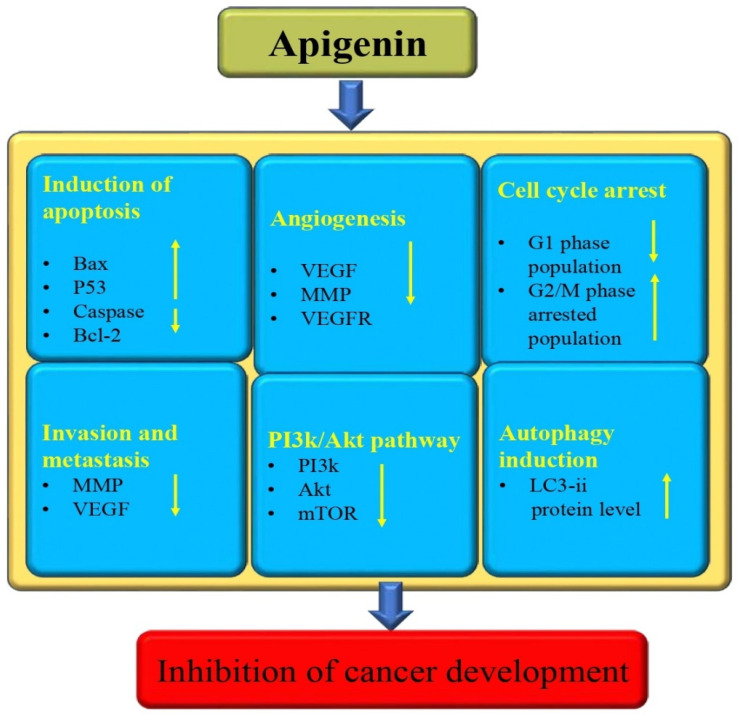
Apigenin modulates cell signaling pathways to manage cancer. The upward pointing arrow signifies upregulation and the downward pointing arrow signifies downregulation.

Unfortunately, cancer is currently the leading cause of death in humans, and it continues to be one of the most significant obstacles to prolonging human life expectancy [125,126]. Cancer treatments, like surgery, chemotherapy drugs, and radiotherapy, are effective but cause serious side effects. Unfortunately, the continued use of chemotherapy drugs has directed the development of tumor resistance, resulting in the gradual loss of efficacy of these drugs [127]. Continuous research and development in the field of medicine have led to a growing interest in exploring the potential of medicinal plants for the discovery of new cytotoxic compounds [128]. Apigenin is a noteworthy player in the management of numerous diseases, including cancer, owing to its ability to modulate different cell signaling molecules [129,130,131,132,133] (Figure 8). The modulation of cell signaling pathways has been reported to have an anti-cancer effect by previous studies through this flavonoid. Several researchers have provided evidence of apigenin’s anti-cancer properties. A study was designed to inspect the effects of apigenin on cell proliferation and the apoptosis of human melanoma cells. To examine the effect of apigenin on melanoma cells cell migration, these cancer cells were treated with different concentrations of apigenin. The migration rates were evaluated after being treated with several concentrations of this flavonoid. The migration rates of A375P cells treated with 0, 50, and 100 µM apigenin were 100%, 53%, and 25%, respectively. The apoptosis rates of cells treated with 0, 50, and 100 µM apigenin were 8.7, 40.3, 59.6%, and 11.6, 38.5, and 47.5%, respectively [134]. An important experiment based on lung cancer reported that apigenin, in addition to suppressing endothelial cell-related motilities, also has the potential to reduce pericyte coverage. Apigenin has demonstrated promising results in reducing microvessel density and pericyte coverage in the xenograft model of NCI-H1299 cells. This innovation has led to the suppression of tumor growth. Also, apigenin has demonstrated a perfect anti-angiogenic effect in the xenograft model of LUSC cell NCI-H1703 cells, making it a likely candidate for development into an effective angiogenesis inhibitor for lung squamous cell carcinoma patients [135]. To examine the effect of apigenin on the viability of hepatocellular carcinoma cells, HepG2 cells were treated with apigenin, and it was noticed that this flavonoid inhibited cell viability in a time- and dose-dependent way. Moreover, apigenin initiated cell death in a dose-dependent way, reduced the expression of Bcl-2, and enhanced cleaved PARP, cleaved caspase-3, cleaved caspase-9, and Bax expression. Moreover, apigenin treatment activated autophagic flux apigenin and enhanced the protein levels of LC3-II in a dose-dependent way in HepG2 cells [136]. Apigenin showed a role in renal cancer cells, as it induced G2/M phase cell-cycle arrest [137]. 

A study based on renal cell carcinoma reported that considerable cell-cycle arrest in the G2/M phase was seen in apigenin-treated cancer cells compared to untreated controls. Cell-cycle analysis findings demonstrated that in treated cells, the G2/M phase-arrested population increased to 16.85% with 5 μM apigenin, 30.54% with 10 μM apigenin, and 46.77% with 20 μM apigenin. Treatment also decreased the G1 phase population. The bladder cancer-based study demonstrated that IL-1β might significantly induce expression of uPAR in these cancer cells and that apigenin-prevented IL-1β could induce expression of uPAR concentration independently. Apigenin can inhibit the expression of uPAR by suppressing the transcriptional activity of both AP-1 and NF-κB through the inhibition of the ERK1/2 and JNK signaling pathways in human bladder cancer T24 cells [138]. This compound, according to reports, can activate the oncosuppressor function of p53 in cancer cells carrying wild-type or mutant p53. An experiment was made to explore the role of apigenin on primary effusion lymphoma (PEL), which is characterized by constitutive activation of STAT3 and other oncogenic pathways, and which harbors wild-type p53. It was observed that p53 silencing in BC3 cells hindered the upregulation of both p21 and catalase following apigenin treatment. Furthermore, p53 silencing also partially prevented apigenin-induced cell death, as evidenced by the results of the viability and PARP cleavage assays [139]. According to a recent study, apigenin can prevent the growth of glioma cells by promoting miR-16. The study concluded that apigenin may have potential anticancer effects against glioma cells [140].

### 3.14. Anti-Arthritis Activity

Natural products and their bioactive compounds have been shown to play a noteworthy role in the management of arthritis through the modulation of various biological activities. These natural products have been found to hold anti-inflammatory as well as immunomodulatory potentials, which can play a role in the management of arthritis. The anti-arthritis potential of apigenin has been reported in previous studies (Table 1). A study was conducted to investigate the impact of macrophage polarization on chondrocyte injury in osteoarthritis, as well as to explore the protective effects of apigenin on chondrocytes. The obtained results from the study, i.e., the histopathological staining, demonstrated that apigenin showed a significant effect on cartilage degeneration. The study also described that apigenin can diminish the expression of TNF-α, IL-6, IL-1, and IL-12 in macrophages, whereas it increases the levels of MG-L1, ARG-1, MG-L2, and IL-10. This can prevent the M1 polarization of macrophages and promote M2 polarization, indicating apigenin’s potential to positively impact macrophage polarization in osteoarthritis [141]. An important study result reported that as compared with the vehicle-treated collagen-induced arthritis mice, the apigenin-treated collagen-induced arthritis animals presented a later onset of collagen-induced arthritis, a reduced incidence of arthritis, and reduced joint swelling. Histological examinations also designated that this compound treatment suppressed synovial hyperplasia as well as inflammatory cell accumulation in the joint space. Moreover, apigenin treatment suggestively reduced the production of the pro-inflammatory cytokines in the supernatant from the LN cells of the collagen-induced arthritis mice. An in vitro study reported that this flavonoid capably controlled the phenotypic as well as functional maturation of LPS-stimulated bone marrow-derived dendritic cells while maintaining phagocytotic capabilities [142]. An apigenin-treated group with a dosage of 40 mg/kg has shown important protection against synovial inflammatory cell infiltration, epithelial cell degeneration, articular cartilage lesion, and synovial tissue edema as compared to the model group. However, the apigenin-treated group with a dosage of 20 mg/kg has shown only mild degeneration of epithelial cells and tissue congestion. Apigenin has meaningfully suppressed the expressions of P2X7/NF-jB signal-associated proteins and has shown positive effects in improving inflammatory reactions [143].

### 3.15. Role in Eye Health

A study result based on in vivo data revealed that apigenin meaningfully reduced the clinical as well as pathological scores of experimental autoimmune uveitis. In the retina, inflammatory cytokines protein levels were decreased, and BRB disruption was improved after apigenin treatment. According to in vitro studies Apigenin has the ability to decrease the production of inflammatory factors in microglia that are induced by LPS as well as IFN-γ [144].

The administration of apigenin resulted in a significant improvement in the recovery of ocular surface function, as well as a decrease in the levels of TNF-α, IL-1β, and IL-6. Additionally, the level of IL-10 was increased with apigenin treatment [145]. Apigenin and ethaverine hydrochloride stabilize endothelial cell junctions and enhance the vascular barrier via increasing VE-cadherin membrane localization and blocking ARF6 activation [146]. The solid dispersion of apigenin (AP-SD) has shown promising results in alleviating retinopathy in a model mouse with dry AMD [147].

### 3.16. Role in Oral Health

Apigenin was given to mouse molar pulp after mechanical pulpal exposure to inspect the detailed function of apigenin in regulating tertiary dentin formation and pulpal inflammation. In vitro-based data show that cultivation of hDPSCs with apigenin treatment enhanced bone morphogenetic protein and osteogenesis-related signaling molecules after 14 days. The specimens treated with apigenin revealed changes in the immunolocalization patterns of certain proteins, such as TNF-α, NESTIN, MPO, and TGF-β1, after 3 and 5 days. Furthermore, the apigenin-treated group exhibited an improved dentin bridge formation with a smaller number of irregular tubules after 42 days of pulpal cavity preparation [148]. A study is being conducted to explore the effects of apigenin on osteoblasts. The study involves incubating hOBs with varying concentrations of apigenin to evaluate cell viability, morphology, and proliferation at different time intervals. This compound displayed a stimulating effect on cell growth, with remarkable and enhanced osteoblast mineralization activities and meaningfully enhanced COL1 and ALP gene expression [149]. A study was conducted in vitro to add apigenin and tt-Farnesol to resin composite and resin cement to decrease the virulence of Streptococcus mutans in the vicinity of dental restorations. In the study, apigenin alone and combined with tt-Farnesol or combined with tt-Farnesol and fluoride were found to decrease the bacterial dry-weight, intracellular polysaccharides, and alkali-solubility [150]. The study examined whether the association of apigenin and tt-farnesol could enhance the anti-caries properties of fluoride. Biofilms treated with apigenin and/or tt-farnesol in combination with fluoride showed less biomass as well as fewer insoluble glucans and iodophilic polysaccharides than those treated with the test agents alone. The combination of the test agents with fluoride was greatly effective in inhibiting caries development in rats, particularly apigenin + tt-Farnesol + fluoride [151].

### 3.17. Radioprotective Effects

The radioprotective potential of apigenin has been reported by previous studies (Table 1). Kanokporn Noy Rithidech and colleagues experimented to investigate the radioprotective and genotoxic effects of apigenin on radiation-induced chromosome aberrations in human lymphocytes. The frequency of micronuclei in irradiated cells increased meaningfully, but the frequency diminished as the concentration of apigenin enhanced, suggesting a radioprotective effect [152]. Another study result revealed that intestinal crypt cells in the apigenin-treated group showed more proliferation and less apoptosis. Additionally, apigenin enhanced the Nrf2 expression and its downstream target gene HO-1 and reduced oxidative stress after irradiation. To summarize, the findings show the radioprotective efficacy of apigenin [153]. The study aimed to investigate whether apigenin could potentially protect against the effects of whole-body gamma irradiation, including oxidative damage and changes in hematological parameters. The study was conducted using Swiss albino mice as a model organism. Results reported that there was decreased antioxidant status and increased LPO level in 7 Gy-irradiated Swiss albino mice. Furthermore, it has been also observed that WBC, RBC count, and Hb content were decreased in irradiated Swiss albino mice. On the other hand, an important restoration of antioxidant status and decrease in LPO and hematological changes were noticed in the apigenin pretreated group [154]. The study aimed to determine the potential of this flavonoid as an antioxidant and its role in preventing radiation-induced oxidative damage in human peripheral blood lymphocytes (HPBL). The finding demonstrated that apigenin treatment meaningfully modulates the loss of ΔΨm and reduces γ-radiation-induced apoptotic incidence in HPBL, and that apigenin treatment meaningfully preserves cellular antioxidant status [155].

### 3.18. Anti-Colitis Effects

Ulcerative colitis is becoming increasingly prevalent and is considered to be one of the primary types of inflammatory bowel diseases. The main features of ulcerative colitis histopathology include alterations in the intestinal mucosal structure. These alterations involve abnormal epithelial cells, the density of colonic crypts, decreased morphology, altered cells of the lamina propria, and immune cell infiltration [156]. The phytoconstituents, such as terpenes, catechins, flavonoids, quinines, alkaloids, anthocyanins, and anthoxanthins, have been found to have anti-inflammatory and antioxidant effects. These compounds can help modulate the expression of pro-inflammatory signals and are considered potential agents for the treatment of this pathogenesis. The anti-colitis potential of apigenin has been reported by previous studies (Table 1). The effects of apigenin on ulcerative colitis were examined by using a dextran sodium sulfate-induced colitis mouse model. Results revealed that apigenin suggestively relieved the intestinal pathological injury, increased mucin secretion and goblet cell quantity, promoted anti-inflammatory cytokines expression, and inhibited the expression of pro-inflammatory cytokines and the activity of MPO in colon tissue. Apigenin efficiently improved dextran sodium sulfate-initiated colitis by balancing the gut microbiome to prevent inflammation and to protect the gut barrier [157]. Another study reported that oral administration of apigenin-7-O-glucoside increased colon length and improved colonic histopathology. In addition, it intensely restored the colonic expression of pro-inflammatory as well as anti-inflammatory mediators, in addition to enhancing the expression of intestinal barrier markers, such as occluding ZO-1, claudin-1, and claudin-3 [158].

Eman E. Shibrya et al. (2023) explored the therapeutic potential of low-dose gamma (γ) irradiation or apigenin treatment in acetic acid-induced ulcerative colitis in rats. Gamma irradiation and apigenin have been found to have a significant impact in ameliorating acetic acid-induced biochemical and histopathological changes. These therapeutic approaches have been observed to effectively restore the colon contents of the investigated biomarkers. They modulated colon weight/length (W/L) ratio, body weight (BW), and disease activity index (DAI). This study concluded that apigenin showed therapeutic benefits in ulcerative colitis management [159]. The objective of the study was to examine the intestinal anti-inflammatory effects of apigenin K, which is a soluble form of apigenin, in two rat colitis models: the trinitrobenzene sulfonic acid model and the dextran sulfate sodium model. Apigenin K pretreatment caused the improvement of morphological signs as well as biochemical markers in this model [160].

In a study, rats were given D. kotschyi hydroalcoholic extract or apigenin orally before being induced with colitis via the intrarectal administration of acetic acid. The dosages for the extract were 10, 20, and 40 mg/kg, while the dosages for apigenin were 5, 10, and 20 mg/kg. According to the study, the activity of MPO increased in the group that was treated with the vehicle; on the other hand, it returned to normal levels when the animals were pretreated with D. kotschyi extract, apigenin, or prednisolone [161].

### 3.19. Wound-Healing Activity

An apigenin-loaded hydrogel was made by utilizing gellan gum–chitosan with PEG as a cross-linker. This formulation of apigenin has demonstrated enhanced wound-healing effects in diabetic and normal wound tissues. Results have proven that the prepared hydrogel, which is loaded with apigenin and GGCH-HGs, has inimitable properties that make it highly appropriate for wound healing [162]. A recent study reported that, based on in vivo study, diabetic mice had meaningfully delayed wound healing as compared to non-diabetic mice and that there was faster wound healing in apigenin-treated diabetic mice [163]. An experiment was carried out to examine the healing effect of topical apigenin cream 2% in the skin of rabbits. The results showed that the group treated with apigenin displayed a meaningfully superior wound healing capacity on the skin of rabbits, and the wound contraction ratio and wound size were superior in the apigenin group [164].

### 3.20. Immunomodulatory Effects

Various natural bioactive compounds, such as triterpenoids, phytosterols, and flavonoids, have been discovered to establish immunomodulatory effects on molecular pathways that show a role in immune responses against various diseases [165]. The immunomodulatory potential of apigenin has been reported by previous studies (Table 1). A recent study evaluated the impact of apigenin on DC phenotypic changes during maturation with LPS. The study found that LPS treatment increased the number of activated DCs that exhibited the expression of various markers. After apigenin treatment, a marked reduction in the level of the antigen presentation molecules HLA-DR and HLA-ABC was noted. On the other hand, the expression of CCR5, a marker of immature DCs, decreases upon LPS-induced maturation and is enhanced to some extent in the presence of apigenin. Following the analysis of the DC cytokine profile in response to apigenin treatment, it was analyzed whether this DC modulation had any impact on the subsequent differentiation of naïve T-cells. DCs were matured in the presence of LPS (2 μg/mL) for 24 h. Apigenin was then administered for 3 h after the end of LPS treatment, and the DCs were plated with PBLs. Th1 polarizing transcription factor T-bet was decreased in PBLs co-cultured with flavonoid-treated mature DCs [166].

**Table 1:** Apigenin for disease management: revealing its multifaceted mechanisms of action.

Activity	Study Type	Dose	Mechanism	Outcome	Refs.
Anti-inflammatory	In vitro	10, 20, 40 Μm	TNF-α ↓, IL-1ß ↓, and IL-6 ↓	Apigenin suppressed these inflammatory markers	[52]
	In vivo	20 and 40 mg/kg	TNF α ↓, IL-1-ß ↓, and IL-6 ↓	The levels of pro-inflammatory cytokines were reduced by apigenin	[55]
	In vitro	30 μM	*IL-10* ↓ and *TNF-α ↓*	Apigenin decreases the expression of *IL-10* and *TNF-α* and secretion	[56]
	In vitro	10, 50 μM	ICAM-1 ↓, COX-2 ↓, VCAM-1 ↓ NO ↓	Apigenin exhibits anti-inflammatory properties by inhibiting the expression of COX-2 facilitated by NO	[61]
Anti-diabetic	In vivo	20 mg/kg	Fibrosis ↓, oxidative stress ↓	Apigenin treatment reduced renal dysfunction as well as oxidative stress It also decreased fibrosis	[69]
	In vivo	0.78 mg/kg	Insulin ↑, glucose ↓	This compound increased the levels of thyroid hormones and serum insulin Decrease in hepatic G-6-Pase and glucose concentration activity This compound decreased LOP and increased antioxidant enzymes	[71]
Hepatoprotective	In vivo	50, 100, 200 mg/kg	Liver function enzyme ↓, oxidative stress ↓, inflammation ↓	Apigenin decrease enzyme function, caused by CCl4 Alleviated the lipid peroxidation as malondialdehyde decreased	[75]
	In vitro	10, 20, and 40 μM	OxidativesStress ↓, inflammation ↓	Apigenin ameliorated oxidative stress Apigenin alleviated H_2_O_2_-induced upregulated expression of pro-inflammatory cytokines	[75]
	In vivo	25 mg/kg	Oxidative stress ↓, fibrosis ↓, inflammation ↓	Apigenin pretreatment prevented oxidative stress, fibrosis, and inflammation	[76]
	In vivo	150–300 mg/kg	Lipid peroxidation ↓ and oxidative stress ↓	Apigenin treatment has been proven to reduce NF-kappa B proteins c and malondialdehyde significantly The levels of hepatic reduced antioxidant enzymes were increased An important change in hepatic steatosis was seen	[78]
Reno protective	In vivo	10, 15, and 20 mg/kg	Lipid hydroperoxides ↓, antioxidant level ↑	Lipid hydroperoxides were reduced and total antioxidant levels were enhanced by apigenin Simultaneous apigenin treatment caused a reduction in the histopathological changes	[82]
	In vivo	20, 40 mg/kg	Infarct size ↓, LDH ↓, CPK ↓	Reducing renal function markers This compound played a role in the upregulation of expressions of ATP6 and ATP8 and relieving the rise in the Bax/Bcl2 ratio	[83]
Cardioprotective	In vivo	50 and 100 mg/kg	Inflammation ↓, nuclear translocation of NF-κB ↓	Myocardial infarct size was reduced by this compound It decreases myocardial injury enzymes and pro-inflammatory cytokines	[86]
	In vitro	1, 6, and 25 μM	Pyroptosis and apoptosis ↓	Pyroptosis and apoptosis stimulated by I/H injury was suppressed by apigenin	[87]
	In vivo	5 mg/kg	LDH activity, intracellular reactive oxygen species generation ↓, improved the loss of mitochondrial membrane potential, antioxidant enzymes ↑	Apigenin protects the myocardium against SI/R injury	[88]
	In vitro	20 μM	LDH activity, intracellular reactive oxygen species generation ↓, Hes1 expression ↑	SI/R injury-induced expression of Hes1 in the cardiomyocytes, which was enhanced in the apigenin plus SI/R cardiomyocytes and decreased by cotreatment of GSI This compound pretreatment maintained mitochondrial function	[88]
Neuroprotective	In vivo	20 and 40 mg/kg	Lucigenin and luminol ↓ and IL-10 levels ↑	Apigenin treatment showed neuroprotective effects via increasing the IL-10 levels and diminishing the level of lucigenin and luminol	[94]
	In vivo	10 and 20 mg/kg	Inflammation and pxidative stress ↓, apoptosis ↓	Apigenin plays a role in promoting the recovery of rat neuronal function	[95]
Role in the respiratory system	In vivo	10, 15, and 20 mg/kg	Fibrosis ↓, antioxidant enzymes ↑, tumor necrosis factor (TNF)-α, and transforming growth factor ↓	Apigenin prevents the fibrotic process The treatment was found to suppress the increases in (TNF)-α levels Attenuated the reduction in superoxide dismutase activity	[101]
	In vivo	20 mg/kg	Expression of NF-κB ↓	This compound showed anti-allergic as well as anti-neutrophil-related inflammatory activity	[102]
Role in the reproductive system	In vivo	25 mg/kg	Sperm density ↓, seminiferous epithelium cells ↑	Sperm density was reduced by apigenin treatment The % of seminiferous epithelium cells was increased	[107]
	In vivo	20 or 40 mg/kg	Testosterone levels and sperm ↑, antioxidant enzymes ↑, and inflammation ↓	The administration of apigenin attenuated testosterone levels as well as sperm measurements This compound administration reduced the CP-induced testicular damage via increasing antioxidants, suppressing the pro-inflammatory cytokines, and decreasing apoptosis	[110]
Role in skin health	In vitro	5, 10, and 20 µM	Cellular senescence ↓ MMP-1 ↓	The number of senescent cells decreased in a dose-dependent manner UVA-induced induction of MMP-1 expression was inhibited by this compound	[111]
	In vivo	Cream containing 1% apigenin	Dermal thickness and skin texture ↑	Topical application of this compound enhances dermal thickness Skin elasticity and hydration were improved	[111]
	In vivo	0.1% apigenin	Transepidermal water loss ↓, skin surface pH ↓, and hydration increased ↑	Application of apigenin topically showed important reductions in transepidermal water loss and skin surface pH, while skin hydration increased	[114]
Anti-obesity	In vivo	0.005% (*w*/*w*) apigenin	Free fatty acid ↓, apolipoprotein B ↓, total cholesterol C ↓	Apigenin has been found to reduce the levels of apolipoprotein B, free fatty acid, and total cholesterol in mice that were fed a high-fat diet	[117]
Anti-depression	In vivo	12.5–25 mg/kg	Antioxidant enzymes ↑, corticosterone and lipid peroxidation ↑	Treatment with apigenin reduced anxiety and immobility time and reversed anhedonia Apigenin increased levels of glutathione and superoxide dismutase and reduced corticosterone and malondialdehyde levels	[121]
	In vivo	20 and 40 mg/kg	Hippocampal brain-derived neurotrophic factor ↑ and corticosterone levels ↓	Apigenin showed the upregulation of brain-derived neurotrophic factor levels	[124]
Anti-cancer	In vitro	0, 50, and 100 µM	Bax, p53 ↑ Bcl2 ↓	The levels of pro-apoptotic proteins and cleaved PARP expression were increased by apigenin This compound downregulated the expression of Bcl-2	[134]
	In vitro	0, 10, 20, and 40 μM	Apoptosis and autophagy ↑, PI3K/Akt/mTOR ↓	Apigenin induced apoptosis, inhibited cell growth, and triggered autophagy Apigenin-induced autophagy as well as apoptosis	[136]
		10, 20, and 40 μM	G2/M phase cell-cycle arrest, Bax, caspases 9 and 3 ↑	Apigenin causes DNA damage and G2/M phase cell-cycle arrest Pro-apoptotic protein and cleaved caspases 9 and 3 increased	[137]
	In vitro	0, 10, 20, and 30 μg/mL	BCL2 and NF-κB/MMP-9 ↓ miR-16 ↑	Apigenin promotes miR-16 expression and induces apoptotic cell death Apigenin inhibits cancer cell growth via suppression of BCL2 as well as NF-κB/MMP-9 and promoting miR-16	[140]
Anti-arthritis	In vivo	20 mg/kg	Pro-inflammatory cytokines ↓, Langerhans cells and Maturation and migration of dendritic cell ↓	Apigenin treatment delayed the onset as well as reduced the severity of arthritis Synovial hyperplasia was suppressed by apigenin treatment	[142]
	In vivo	20 and 40 mg/kg	Inflammation ↓ P2X7/NF-κB signal-related proteins ↓	This compound protects against synovial inflammatory cell infiltration, synovial tissue edema, and epithelial cell degeneration Apigenin treatment caused mild degeneration of epithelial cells as well as tissue congestion	[143]
Role in eye-associated pathogenesis	In vivo and in vitro	20 mg/kg and 20 and 40 μM	Inflammatory cytokines ↓, BRB disruption ↓, microglial M1 polarization inhibited	Apigenin treatment ameliorated the manifestations of disruption of the BRB Inflammatory cytokines protein were levels decreased by this compound Microglial M1 polarization via TLR4/MyD88 signaling was inhibited by apigenin	[144]
	In vivo	10, 20, and 50 mg/kg	Inflammation ↓	Apigenin treatment in dry-eye disease decreased the levels of TNF-α, IL-1β, and IL-6	[145]
Role in dental-associated pathogenesis	In vitro and in vivo	5 μM and 50 μM	Bone morphogenetic protein and osteogenesis-related signaling molecules ↑, TNF-α ↓	The highest ALP activity was noticed with apigenin treatment Bone morphogenetic protein-related as well as osteogenesis-related signaling molecules are enhanced by apigenin-treatment The localization of TNF-α is diminished in apigenin-treatment	[148]
Radioprotective	In vitro	2.5, 5, 10, and 25 µg/mL	Cytokinesis-block proliferation index ↓, cell proliferation ↓	The cytokinesis-block proliferation index was decreased after the apigenin concentration increased A severe decrease in cell proliferation was seen in cells treated with this compound	[152]
Anti-colitis	In vivo	3 mg/kg/day	MDA and NOx contents ↓, MPO activity ↓, mucosal addressin cell adhesion molecule-1 ↓, IL-1β content ↓	Apigenin improved the acetic acid-induced biochemical and pathological changes This compound maintained the colon contents of the studied biomarkers	[159]
	In vivo	10, 20, and 40 mg/kg	MPO activity ↓ inflammation ↓	This compound acts as an anti-inflammatory potential	[161]
Wound healing	In vivo	Apigenin cream 2%	wound size ↓ and wound contraction ↑	Superior wound healing capacity, wound contraction ratio, and wound size were noted in the animal treated with apigenin	[164]
Immunomodulatory	In vitro	1–20 μM	mRNA and protein levels of RelB ↓, pro-inflammatory cytokine production ↓	Apigenin showed a role in the reduction of soluble TNF-α release Apigenin impairs dendritic cells’ phenotypic as well as functional maturation	[166]

### 3.21. Anti-Microbial Activity

Apigenin plays a substantial role in killing or minimizing the action of microorganisms in different ways. It can be a valuable means of fighting against harmful pathogens with antibacterial, antiviral, antifungal, and anti-parasitic potential (Table 2). The anti-microbial activity of this compound is discussed as follows.

i. Antibacterial activity

The study examined the role of pure apigenin on human gut bacteria, at both the single-strain and community levels. When cultured alone, *Enterococcus caccae* was efficiently prevented by apigenin. It was revealed that *Enterococcus caccae* responds to apigenin by upregulating genes associated with stress response, DNA repair, cell wall synthesis, and protein folding [167]. An experiment was conducted to explore the effects of apigenin and tt-farnesol, alone as well as in combination, on the accumulation, polysaccharide composition, and viability of *S. mutans* UA159 biofilms. It is interesting to note that the fructosyltransferase activity was only affected by apigenin and apigenin + tt-farnesol and that the recoverable viable counts of *S. mutans* were slightly lower after apigenin and tt-farnesol treatments compared to the vehicle control [168]. Apigenin was found to reverse bacterial resistance to cephalosporin ceftazidime in Enterobacter cloacae. The identification of the 5,7-OH group of the A ring and one 4′-OH group of the B ring in apigenin being important in reversing antimicrobial resistance is also noteworthy [169]. The results indicate that the combination of ampicillin as well as ceftriaxone with apigenin was directed to damage the cytoplasmic membrane of MRSA and caused the leakage of intracellular components [170], and apigenin causes bacterial apoptosis via the activation of cellular oxidative pathways [171].

**Table 2:** Apigenin as an antimicrobial agent.

Activity	Species	Mechanism	Outcome	Refs.
Antibacterial	*Enterococcus caccae*	Alters the gene expression; membrane synthesis and protein misfolding are affected	Growth and gene expression of Enterococcus caccae was affected by this compound	[167]
	*Streptococcus mutans*	Polysaccharide content affected	Apigenin as well as tt-farnesol affected the accumulation and polysaccharide content of *S. mutans* biofilms The fucosyltransferase activity was affected only by apigenin	[168]
	*Enterobacter cloacae*	Leakage of intracellular constituents and damage of the CREC cytoplasmic membrane	Apigenin exhibited inhibitory effects against penicillinase type IV The combined application of ceftazidime and apigenin resulted in a significant alteration of outer membrane permeabilization	[169]
	*Staphylococcus aureus*	Leakage of intracellular constituents and damaged MRSA cytoplasmic membrane	The combination of ampicillin and ceftriaxone with apigenin caused cytoplasmic membrane damage of MRSA and led to the leakage of intracellular constituents The combined use of ampicillin and ceftriaxone with apigenin resulted in noticeable morphological damage to the cell wall, cell shape, and plasma membrane	[170]
	*Escherichia coli*	Increase in intracellular calcium and induces bacterial apoptosis	Bacterial apoptosis was caused by apigenin by the activation of cellular oxidative pathways	[171]
Antifungal	*Candida albicans*	Morphological changes, cell shrinkage membrane disturbances	Antifungal activity of apigenin was noted via inducing membrane disturbances, which causes cell shrinkage	[172]
	*Candida albicans*	Induced uptake of calcium and induced apoptosis activation	Apigenin triggers various biochemical responses that contribute to its antifungal activity	[173]
Antiviral	*EV71*	Suppressing viral IRES, modulating cellular JNK pathway	Apigenin inhibits EV71 replication	[174]
	*Influenza A virus*	Inhibiting upregulation of retinoic acid-inducible gene-I expression and interferons and pro-inflammatory cytokines	Apigenin inhibits influenza A virus (IAV)-induced inflammation as well as viral replication	[175]
	*EV71*	Disrupting the viral RNA’s suppressed the expression of GFP	Apigenin can selectively block EV71 infection	[176]
Anti-parasitic	*Leishmania amazonensis*	Inhibition of cellular proliferation increased reactive oxygen species generation	Treatment with apigenin caused by concentration-dependent inhibition of cellular proliferation	[177]

ii. Antifungal activity

Fungi can lead to a range of illnesses in humans, from allergic reactions to superficial as well as potentially life-threatening invasive fungal diseases, affecting over one billion people globally [178,179]. Resistance to antifungal treatments for infections has been on the rise in recent years. This increase has had significant implications for medical care, morbidity, and mortality in the community, as noted by Arif et al. in 2009 [180]. Natural products and their bioactive compounds play a substantial role in the control of fungal diseases. The study aims to identify and isolate a specific compound from the stem extract of Terminalia chebula, which is the most active extract in treating dermatophytosis in experimental mice. According to the study, the reference drug terbinafine and apigenin II ointment with a concentration of 5 mg g(−1) resulted in complete recovery from the infection on the 12th day of treatment. On the other hand, the apigenin I ointment with a concentration of 2.5 mg g (−1) showed a complete cure on the 16th day of treatment. Based on the study’s results, apigenin can be explored as a potential antifungal agent for the clinical treatment of dermatophytosis in the future [148,181]. Antifungal activity as well as a mode of action of apigenin were examined. Apigenin prevented the growth of fungal pathogens, which resulted in reduced biofilm mass and superficial infection [172]. A study was performed to explore the biochemical responses underlying the antifungal potential of apigenin isolated from A. yomena. Apigenin, which was isolated from A. yomena, triggered various biochemical responses that contribute to its antifungal activity. Specifically, the compound induced a dose-dependent uptake of calcium by the mitochondria, which, in turn, caused mitochondrial dysfunction [173].

iii. Antiviral activity

Apigenin has a proven role in controlling viral pathogenesis through certain mechanisms. A study reported that apigenin prevented EV71-induced c-Jun N-terminal kinase (JNK) activation, which is important for viral replication and this compound inhibits EV71 replication by suppressing viral IRES activity as well as by modulating the cellular JNK pathway [174]. Another study was performed to explore the role of apigenin on influenza A virus (IAV)-induced inflammation as well as viral replication. This study confirmed that apigenin treatment meaningfully suppressed IAV-induced upregulation of retinoic acid-inducible gene-I (RIG-I) expression [175]. A recent study result reported that apigenin protected embryonated eggs from the infectious bronchitis virus. Furthermore, apigenin decreases the log titer of the infectious bronchitis virus, with a significant correlation of up to 9.4 times at 2 µg/egg. Similarly, this compound appears to meaningfully reduce the infectious bronchitis virus genome copy number (per µL) in the allantoic fluid [182]. It was noticed that apigenin has the ability to selectively block EV71 infection [176].

iv Anti-parasitic activity

Anti-parasitic effects of apigenin have been shown by previous studies. A concentration-dependent inhibition of the *L. infantum* promastigote in the presence of apigenin was noted, showing an IC_50_ value of 29.9 µM. In a murine model of visceral leishmaniasis, the in vivo potential of apigenin was evaluated via short-term and long-term treatment schemes. Apigenin treatment showed a 99.7% and 94% decrease in the liver parasite load in the short-term as well as long-term treatment schemes, respectively [183].

The antileishmanial potential of apigenin in vitro was evaluated, and its mechanism of action against Leishmania amazonensis promastigotes was explained. Treatment with apigenin for 24 h caused a concentration-dependent inhibition of cellular proliferation as well as increased reactive oxygen species generation [177].

## 4. Synergistic Effects of Apigenin with Drugs/Natural Compounds

The modulation of biological activities and cell signaling molecules confirms apigenin’s role in managing diseases including cancer. Its synergistic effects have been reported with other natural compounds and drugs (Table 3). Experimental evidence suggests that the combination of this compound with other drugs has the potential to increase the activity of the drugs while reducing treatment resistance. The combined use of ampicillin and ceftriaxone, along with apigenin, has demonstrated synergistic activity with FIC indices (CI) ranging from 0.18–0.47. It is noteworthy that the time-kill assays for the two drug combinations—ampicillin + apigenin and ceftriaxone + apigenin—have shown a significant inhibitory effect against MRSA [170]. The study examined the latent synergistic antiproliferative functions of apigenin and naringenin in non-small cell lung cancer. The results recognized that the combination of apigenin and naringenin (CoAN) treatment caused noteworthy cytotoxicity with cell-cycle arrest at G2/M phases. Additionally, the combination of Api and Nar (CoAN) meaningfully enhanced mitochondria dysfunction, activated the apoptotic pathway, and elevated oxidative stress, versus apigenin or naringenin only groups [184]. The study aimed to explore the potential anti-inflammatory effects of apigenin and curcumin co-nanoencapsulated in sodium caseinate, in comparison with unencapsulated polyphenol combinations. Combinations of free polyphenols showed better inhibition of nitric oxide production, more noteworthy at a higher proportion of curcumin, which was further increased after co-encapsulation [185].

A combination of two powerful chemotherapeutic agents, namely liposomal apigenin and tyroservatide, was used to treat lung cancer. The role of this combination was evaluated by studying its effects on lung cancer cells. Significantly, when compared to either agent alone, the combination showed greater cytotoxicity, brought superior G2 arrest, and suppressed the invasion of these cancer cells. In vivo, the results showed that the combination form displayed tumor-growth inhibitory potential [186]. Another study result reported that apigenin and myricetin, when combined, have a positive effect on reducing blood BUN, COXI, IL-6, serum Cr COXII, and MDA levels in addition to enhancing GSH levels as well as catalase activity. Cisplatin-induced nephrotoxicity was linked with enhanced serum caspase-3 activity, demonstrating apoptosis of renal cells. Pretreatment with myricetin, apigenin, or their combination meaningfully decreased serum caspase-3 activity as compared with the cisplatin-treated group [187]. Another recent study result reported that a combination of curcumin and apigenin exhibited a synergistic antitumor effect through cross-talk between processes causing apoptosis as well as autophagic cell death, and stress-associated paraptosis. GRP78 expression was decreased, and huge cytoplasmic vacuolization was seen in HeLa cells [188]. A study based on prostate cancer reported that a combination of abiraterone acetate and apigenin meaningfully inhibited LNCaP and androgen-insensitive PC3 cell survival in a manner more noticeable than was seen with either single treatment [189]. Apigenin and curcumin have a synergistic effect in fighting cancer. They were found to induce cell death and apoptosis, as well as to block cell-cycle progression in lung cancer cells specifically at the G2/M phase. The synergistic activity of apigenin and curcumin may be due to their effects on microtubules. Studies have shown that these natural compounds have strong depolymerizing effects on interphase microtubules and can inhibit the reassembly of cold depolymerized microtubules when used in combination [190]. In studies on hepatocellular cancer cells, it was found that the combination of sorafenib and apigenin resulted in a more significant decrease in cell viability than either treatment alone [191].

**Table 3:** Synergistic effects of apigenin with other drugs/natural compounds.

Apigenin	Compound/Drugs	Study Type	Outcome	Refs.
Apigenin	Ampicillin and ceftriaxone	In vitro	The combined use of ampicillin and ceftriaxone, along with apigenin, has demonstrated synergistic activity with FIC indices ranging from 0.18–0.47 The combination of ampicillin and ceftriaxone, along with apigenin, can damage the cytoplasmic membrane of MRSA	[170]
	Naringenin	In vitro	The combination of apigenin as well as naringenin treatment caused cytotoxicity with cell-cycle arrest Combination treatment activated the apoptotic pathway and enhanced mitochondria dysfunction versus apigenin-only groups	[184]
	Curcumin	In vitro	The co-nanoencapsulation of curcumin and apigenin shows applications in utilizing their synergistic anti-inflammatory properties Combinations of free polyphenols showed good inhibition of nitric oxide production, which was additionally increased after co-encapsulation	[185]
	Tyroservatide	In vitro and in vivo	The combined form displayed inhibitory tumor-growth potential	[186]
	Myricetin	In vivo	Apigenin and myricetin, when combined, have a positive effect on reducing IL-6, MDA, blood BUN, and serum Cr levels and enhance antioxidant enzyme activity Renal tissue from cisplatin-treated mice pretreated combination showed fewer renal changes	[187]
	Curcumin	In vitro	Combination of curcumin and apigenin exhibited a synergistic antitumor effect	[188]
	Abiraterone acetate	In vitro	Co-administration of apigenin with abiraterone acetate prompted apoptosis	[189]
	Curcumin	In vitro	Combination induces cell death and apoptosis, as well as block cell-cycle progression in lung cancer cells	[190]
	Sorafenib	In vitro	Combined treatment of sorafenib and apigenin decreased cell viability as compared to single treatment only	[191]
	Paclitaxel	In vitro	This compound can sensitize cancer cells to paclitaxel-induced apoptosis	[192]
	Etoposide or cyclophosphamide	In vitro	Combining apigenin with etoposide or cyclophosphamide triggers apoptosis This is achieved by increasing the expression of pro-apoptotic factors, which in turn promotes the activation of caspase-9 and -3	[193]
	Cisplatin	In vitro	When apigenin is combined with etoposide or cyclophosphamide, it can trigger apoptosis through the mitochondrial pathway. This leads to the phosphorylation of p53, which in turn results in p53 accumulation as well as upregulation of proapoptotic proteins	[194]
		In vitro	Apigenin repressed CD 133 positive cells and enhanced the antitumor effect of CDDP in cancer cells.	[195]

## 5. Nanoformulation of Apigenin and Pharmacological Action

Apigenin has been found to have numerous health benefits and is known for its antioxidant and anti-inflammatory properties, as well as its ability to manage cancer. Apigenin achieves these benefits by modulating various biological pathways in the body. However, despite its many health benefits, apigenin’s potential effectiveness may be hindered by low absorption, poor solubility, and fast elimination and metabolism. These factors can all contribute to poor bioavailability, making it difficult for the body to fully utilize the compound for its health-promoting effects.

The clinical development of apigenin has been hindered by its low solubility in both lipids and water, which results in a relatively low oral bioavailability. According to published studies, it has been confirmed that apigenin has a low solubility in both fat and water. Its solubility is 2.16 μg/mL in water and 0.001–1.63 mg/mL in non-polar solvents [196], while its water solubility is only 2.16 μg/mL [197]. A study found that the plasma concentrations of apigenin peaked at 3.9 h after dosing. Additionally, the total recovery of the dose was 45.2%, with 16.6% found in urine and 28.6% in feces for apigenin, and the cumulative excretion of apigenin was 6.34% [198]. A study was conducted to test the absorption and excretion of apigenin after the ingestion of apiin-rich food, i.e., parsley. On average, it takes 7.2 ± 1.3 h to reach a maximum apigenin plasma concentration of 127 ± 81 nmol/L. After ingesting the bolus, the plasma concentration of apigenin increased in all participants, then decreased below the detection limit of 2.3 nmol/L within 28 h [32]. Researchers investigated the bioavailability of apigenin and its O-glycosides in humans. Consuming a parsley drink containing apigenin-7-O-(2″-O-apiosyl) glucoside resulted in the peak plasma concentration (Cmax) of Ap-4′-GlcUA occurring after 4 h, which indicates absorption in the lower gastrointestinal tract (GIT). When dried powdered parsley leaves were ingested with yogurt, the Cmax of Ap-4′-GlcUA extended to 6 h. Consuming chamomile tea containing apigenin-7′-O-glucoside resulted in a 2 h Cmax of Ap-4′-GlcUA, indicating absorption in the upper gastrointestinal tract (GIT) [199].

**Figure 9:**
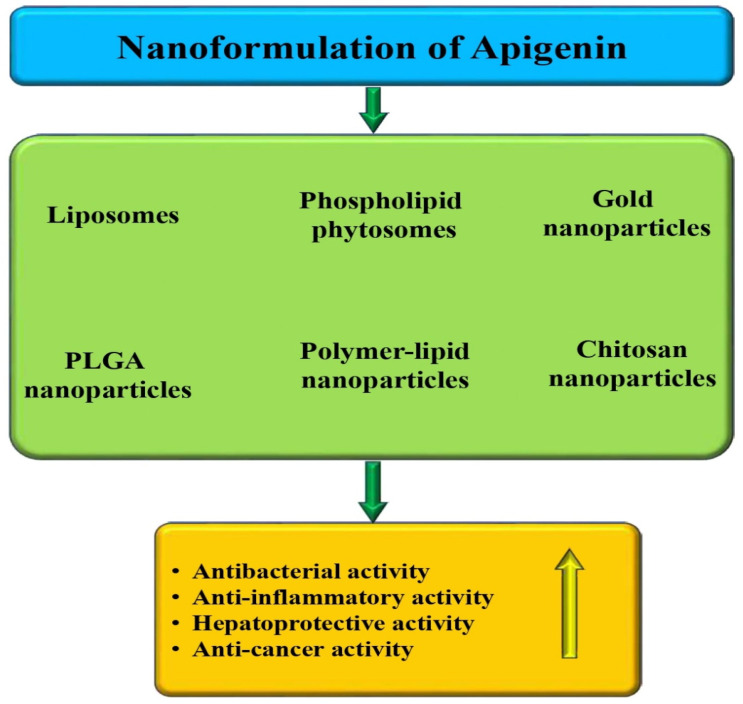
Nanoformulations and their role in different pathogenesis assessed. The upward pointing arrow indicates upregulation.

To address the issues with apigenin’s bioavailability, experimentations have turned to nanotechnology-based approaches. These methods have been used to enhance the apigenin’s ability to contribute to disease management and to improve its bioavailability. Conventional cancer treatment methods often have several drawbacks, such as causing damage to healthy tissues, leading to serious side effects, and potentially contributing to the development of multidrug resistance. It has been increasingly recognized that many natural products, such as curcumin, quercetin, sulforaphane, and berberine, may have promising anticancer activity by specifically targeting cancer stem cells [200,201]. It is worth noting that nanoformulations also have the potential to improve the efficacy of cancer treatment by enabling targeted drug delivery and site-specific release. By encapsulating drugs within nanoformulations, it is possible to achieve improved therapeutic effects while minimizing the risk of side effects caused by high-dosage administration [202]. Indeed, nanoformulation approaches have shown tremendous potential in improving the pharmacokinetic and pharmacodynamic properties of bioactive plant compounds and helping to overcome their inherent limitations. These approaches can enhance the solubility, bioavailability, stability, and targeted delivery of bioactive plant compounds [203,204,205], which could lead to better therapeutic outcomes and reduced side effects. Different types of nanoformulations have been made based on apigenin and its effects on different pathogenesis have been assessed (Table 4 and Figure 9).

A study was performed to explore the antibacterial activity of free and liposomal formulations of apigenin and to clarify the mode of action. Interaction studies showed adherence as well as fusion of liposomal apigenin, with the bacteria causing membrane perturbation via reactive oxygen species generation. The interaction between apigenin liposomes and bacterial membranes increased the intracellular drug concentration. This liposomal apigenin property can be utilized to deliver apigenin within cells to enhance its antibacterial activity [206]. Two oil-in-water formulations, comprising equal amounts of apigenin-enriched chamomile flower extracts, for probable use as topical anti-inflammatory agents, were made. The liposomal formulations used in the study were found to be more viscous, which resulted in superior release characteristics in vitro [207].

An apigenin–phospholipid phytosome was made to improve the dissolution, aqueous solubility, and in vivo bioavailability of the compound. According to pharmacokinetic analysis, the prepared formulation showed a significant improvement in the oral bioavailability of apigenin when compared to pure apigenin [208]. The study explored the cardioprotective effect of gold nanoparticles (AuNPs) synthesized with apigenin (Api) in doxorubicin (DOX)-induced cardiotoxicity (DIC). The results revealed that api-AuNPs therapy substantially reduced body and heart weight loss compared to the DOX group. Injury indicators were reduced dramatically by Api-AuNPs treatment [209]. A study developed gold nanoparticles (AuNPs) by reducing gold salts with apigenin. Recent studies have revealed that api-AuNPs can upregulate the expression of Bid, Bax, and caspase 3, whereas downregulating Bcl2, leads to increased caspase 3/7, 8, 9 activities [210]. Apigenin-loaded nanoparticles showed a sustained drug release pattern as well as effectively reaching the hepatic cancer cells in vitro and in the liver of carcinogenic animals. The use of apigenin-loaded nanoparticles (ApNp) may have a positive impact on hepatocarcinogenesis in rats. ApNp was able to delay the progress of HCC in these rats [211]. Lipid polymer hybrid nanoparticles of apigenin (LPHyNPs) were prepared, and their efficacy in terms of apoptosis as well as cell-cycle arrest against colon cancer was examined. Furthermore, to support the anticancer potential of LPHyNPs against chemoresistance, the expression of JNK as well as MDR-1 genes was assessed. The results showed that their expression level decreased noticeably when compared to blank LPHyNPs and an apigenin suspension [212]. The gold nanoparticles (AuNPs) were made using reducing gold salts with apigenin, and it was reported that upon interacting with lung cancer cells, the nanoparticles were able to cause cell apoptosis, prevent cell proliferation, and arrest cancer cells in G0/G1 phases in a dose-dependent way [213]. Apigenin-loaded polymer–lipid hybrid nanoparticles (AGN-PLHNPs) were prepared. The optimized AGN-PLHNPs showed a greater %RSA as compared to free apigenin. At the concentration of 100 µg/mL, the optimized nanoformulation and free apigenin showed %RSA values of 89.51 ± 4.83% and 54.72 ± 3.67%, respectively. Overall, the optimized AGN-PLHNPs showed meaningfully enhanced %RSA values when compared with free apigenin. The blank PLHNPs showed no toxicity against breast cancer cells, and it can be inferred that the PLHNPs alone are non-toxic as well as safe for drug delivery. Then again, the optimized AGN-PLHNPs and free apigenin showed concentration- and time-dependent cytotoxicity against both breast cancer (MCF-7 and MDA-MB-231) cells [214]. Apigenin-Mn(II)-loaded sodium hyaluronate nanoparticles (API-Mn(II)@HA NPs) were prepared and showed a diameter of 200 nm; they were effective against ulcerative colitis. It was reported that API-Mn(II)@HA nanoparticles have shown promising results in repairing the intestinal barrier and improving damaged colon tissue by regulating inflammatory factors [215]. Dual nanostructured lipid carriers (NLC) encapsulating apigenin (APG-NLC) with a lipid matrix containing rosehip oil, which is identified for its antioxidant and anti-inflammatory properties, were prepared. According to the in vitro studies, APG-NLC exhibited significant anti-angiogenic activity in ovo and selective antiproliferative activity in various cancer cell lines, without showing any toxicity in healthy cells [216]. Apigenin-loaded Cs/Gel membranes were made. The Saos-2 osteoblasts were cultured on membranes to examine the cell–membrane interaction, viability, proliferation, and mineralization under the osteogenic culture condition. Based on the findings, study data suggested that Cs/Gel membranes loaded with low apigenin contents could enhance the survival, proliferation, and mineralization of osteoblasts [217]. The newly developed encapsulated Ap (Ap-CH-BSA-FANPs) was characterized as well as tested in vitro. The in vitro results demonstrated the great anticancer activity of the encapsulated apigenin on HePG-2 cells compared to pure apigenin. The treated HePG-2 cells with Ap-CH-BSA-FANPs established the induction of apoptosis via arresting the cell-cycle, increasing p53 gene expression, increasing caspase-9 levels, and decreasing both the MMP9 gene as well as *Bcl-2* protein expression levels [218].

**Table 4:** Apigenin-based nanoformulation and its role in different pathogenesis.

Nanoformulation Types	Study Types	Outcomes of the Study	Refs.
Liposomal formulation	In vitro	The interaction of apigenin liposomes with bacterial membrane increased intracellular drug concentration	[206]
Liposomal formulation	In vitro and in vivo	Liposomal formulations showed superior characteristics in vitro This formulation showed, to some extent, better in vivo anti-inflammatory activity than their respective non-liposomal counterparts	[207]
Apigenin–phospholipid phytosome	In vitro and in vivo	Rate as well as amount of in vitro apigenin release was increased when compared to the suspension of pure apigenin The prepared phytosome exhibited improved restoration of CCl4-elevated rat liver function marker enzymes	[208]
Apigenin-coated gold nanoparticles	In vitro and in vivo	This formulation was non-toxic to H9c2 heart cells Formulation therapy considerably reduced body and heart weight loss Injury indicators were decreased intensely by formulation treatment	[209]
Apigenin-coated gold nanoparticles	In vitro	The formulation inhibited the migration of cancer cell	[210]
Apigenin-loaded PLGA-nanoparticles	In vitro and in vivo	Formulation showed an IC50 value much lower than that of apigenin. In HepG2 cells, the IC50 value of formulation was 5.5 µg/mL and for apigenin it was 11 µg/mL In the case of Huh-7 cells, the IC50 value of formulation was 8.5 µg/mL and for apigenin it was 12 µg/mL This nanoformulation predominantly delayed the progress of liver cancer	[211]
Lipid–polymer hybrid nanoparticles	In vitro	The formulation has shown promising potential to induce cell-cycle arrest	[212]
Apigenin–gold nanoparticles	In vitro	The prepared nanoparticles induce cell apoptosis and inhibit cell proliferation	[213]
Apigenin-loaded polymer–lipid hybrid nanoparticles	In vitro	The prepared nanoformulation showed enhanced cytotoxicity efficacy against breast cancer cell lines	[214]
Apigenin-Mn (II) loaded sodium hyaluronate nanoparticles	In vivo	Nanoparticles efficiently repair the intestinal barrier and seem to improve the damaged colon tissue	[215]
Apigenin-loaded nanostructured lipid carriers	In vitro	The nanoformulation that has been prepared shows antiangiogenic activity and selective antiproliferative activity in multiple cancer cell lines	[216]
Apigenin-loaded chitosan/gelatin membranes	In vitro	The use of Cs/Gel membranes containing small amounts of apigenin has been found to boost the survival, proliferation, and mineralization of osteoblasts	[217]

## 6. Conclusions and Future Prospective

Apigenin is an essential flavonoid with powerful anti-inflammatory and antioxidant properties. Its efficacy in various pathogenic conditions is well established by modulating biological activities. The ability of apigenin to scavenge free radicals and reduce oxidative stress is its main characteristic in disease prevention. Moreover, the anti-inflammatory potential of this compound plays a vital role in the control of disease processes through the regulation of pro-inflammatory cytokines. Furthermore, its anti-cancer potential has been evidenced based on in vivo and in vitro studies through the modulation of cell signaling molecules. 

Its synergistic effects with other natural compounds and drugs have been reported through modulating various biological activities. Experimental evidence suggests that the combination of this compound with other drugs has the potential to increase the activity of the drugs while reducing treatment resistance.

Despite its role in health-promoting effects, proper clinical development of apigenin has been hindered by its low solubility in both lipid and water, resulting in a relatively low oral bioavailability. Researchers have turned to nanotechnology-based approaches to address the challenges with apigenin’s bioavailability. These methods have shown promise in enhancing apigenin’s disease management capabilities and bioavailability. 

This extensive review based on the impact of apigenin on different pathogeneses and its mechanisms of action is novel and provides valuable information for health researchers. Exploring the various effects of this flavonoid on different diseases and explaining its underlying mechanisms can contribute to advancements in addressing pathogenesis and improving health outcomes. Further research is being conducted to develop different forms of nanoformulations that can increase the efficiency of apigenin and improve its role in health management. Additional clinical trials are needed to explore the potential of apigenin in disease management by understanding this compound’s efficacy, safety, and exact mechanism of action.

## List of Abbreviations

ROS	Reactive oxygen species
API	Apigenin
AGEs	Advanced glycation end products
DOX	Doxorubicin
MDA	Malondialdehyde
IL	Interleukin
TNF-α	Tumor necrosis factor-α
COX-2	Cyclooxygenase-2
MAPK	Mitogen-activated protein kinase
SOD	Superoxide dismutase
CCl4	Carbon tetrachloride
CAT	Catalase
GSH	Glutathione
GPx	Glutathione peroxidase
*ALT*	Alanine transaminase
TBI	Traumatic brain injury
OVA	Ovalbumin
HFD	High-fat diet
VEGFs	Vascular endothelial growth factors
MMPs	Matrix metalloproteinases
BW	Body weight
MRSA	Methicillin-resistant Staphylococcus aureus
MPO	Myeloperoxidase
AuNPs	Gold nanoparticles
ApNp	Apigenin-loaded nanoparticles
VCAM-1	Vascular cell adhesion protein 1
NPs	Nanoparticles
